# Mechanosensitive Ion Channels: Their Physiological Importance and Potential Key Role in Cancer

**DOI:** 10.3390/ijms241813710

**Published:** 2023-09-05

**Authors:** Álvaro Otero-Sobrino, Pablo Blanco-Carlón, Miguel Ángel Navarro-Aguadero, Miguel Gallardo, Joaquín Martínez-López, María Velasco-Estévez

**Affiliations:** 1 H12O-CNIO Hematological Malignancies Clinical Research Unit, Centro Nacional de Investigaciones Oncologicas (CNIO), 28029 Madrid, Spain; aotero@cnio.es (Á.O.-S.); pblanco@cnio.es (P.B.-C.); manavarro@ext.cnio.es (M.Á.N.-A.); mgallardod@cnio.es (M.G.); jmarti01@med.ucm.es (J.M.-L.); 2 Department of Hematology, Hospital Universitario 12 de Octubre, Instituto de Investigacion Sanitaria Hospital 12 de Octubre (imas12), 28041 Madrid, Spain; 3 Department of Medicine, School of Medicine, Universidad Complutense de Madrid (UCM), 28040 Madrid, Spain

**Keywords:** mechanoreceptors, ion channels, TRP, PIEZO, immunity, cancer

## Abstract

Mechanosensitive ion channels comprise a broad group of proteins that sense mechanical extracellular and intracellular changes, translating them into cation influx to adapt and respond to these physical cues. All cells in the organism are mechanosensitive, and these physical cues have proven to have an important role in regulating proliferation, cell fate and differentiation, migration and cellular stress, among other processes. Indeed, the mechanical properties of the extracellular matrix in cancer change drastically due to high cell proliferation and modification of extracellular protein secretion, suggesting an important contribution to tumor cell regulation. In this review, we describe the physiological significance of mechanosensitive ion channels, emphasizing their role in cancer and immunity, and providing compelling proof of the importance of continuing to explore their potential as new therapeutic targets in cancer research.

## 1. Introduction

Mechanosensation is the ability of cells to recognize mechanical and physical forces, and it is essential for many physiological functions, playing a pivotal role in both health and disease. In line with this, it has been shown that there are dramatic intratumoral mechanical changes at the onset of many types of cancer [1]. Cancer cells are constrained by high mechanical cues due to the increased extracellular matrix (ECM) stiffness and the high interstitial pressure of the tumor. Such forces can stimulate the proliferation, migration, and even invasion of cancer cells through mechanosensitive ion channels, thus playing an important role in both the onset and progression of the tumor [2,3].

Mechanosensitive ion channels are a family of pore-forming proteins crucial for detecting intra- and extracellular mechanical cues (e.g., pressure, stretch or shear flow). These channels then translate mechanical cues into biochemical signals in a process termed mechanotransduction, allowing the cell to adapt and respond to mechanical forces [4,5]. Upon mechanical stimuli, the cation influx into the cytoplasm can mediate a myriad of cell responses such as cell growth, migration, adhesion, morphogenesis, gene expression, fluid homeostasis and vesicular transport [6].

Understanding the role of mechanoreceptors in the different cancer types, as well as in the immune system and cancer immunity, is vital to fully uncovering the importance of the mechanical properties of the tumor environment.

## 2. Classification of Mechanoreceptors and Their Physiological Importance

Árnadottir and Chalfie defined the criteria for ion channels to act as mechanoreceptors [7]. First, the channel must be expressed in a mechanosensory organ. Second, the loss of the channel is necessary but insufficient to end the signal transduction. Third, the genetic modification of the channel should change the mechanical response. Lastly, channel heterologous expression must reveal that it can be physically gated. However, new evidence about mechanosensitive ion channels has redefined these criteria and established four different families of mechanoreceptor channels in mammals [8]: epithelial sodium channel/degenerin (ENaC/DEG), transient receptor potential channel (TRP), two-pore domain potassium channel (K2P) and PIEZO channels.

### 2.1. The Epithelial Sodium Channel/Degenerin Superfamily

**Figure 1:**
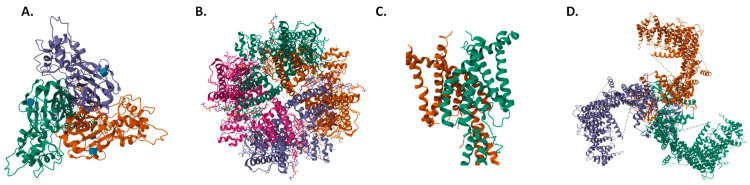
Top view structures of (**A**) hASIC1a [46], (**B**) hTRPV4 [47], (**C**) hTRAAK [48], and (**D**) mPiezo1 [41]. Structures obtained from the Protein Data Bank (PDB IDs 7CFS, 8T1B, 3UM7 and 5Z10), respectively.

The ENaC/DEG family represents a class of ion channels that are present in animals with specialized organ functions and have functional heterogeneity depending on their tissue distribution. In humans, ENaC and acid-sensing ion channels (ASIC) can be found inside this family [9]. Both have a trimeric structure with two transmembrane segments and a large extracellular region [10] (Figure 1A), but they display different gating mechanisms [10,11].

ENaC channels are encoded by four paralogous genes (*SCNN1A*, *SCNN1B*, *SCNN1D* and *SCNN1G*) that encode the subunits α, β, γ and δ, respectively [12]. These channels are expressed on the apical plasma membrane of epithelial cells in several organs, such as the kidney, lung, salivary glands, skin, placenta and colon, and play a central role in Na^+^ absorption, maintenance of water-salt homeostasis, and blood pressure control. Depending on the tissue where ENaC is expressed, its physiological role varies. For instance, in the kidney, filtered Na^+^ exits the collecting duct’s urinary space through ENaC, restricting the amount of salt that can be reabsorbed [13]; in the colon, ENaC also participates in Na^+^ reabsorption [14], whereas in the lungs, ENaC is a key regulator of airway surface liquid clearance [15].

On the other hand, ASICs are primarily proton-gated cation channels that can be triggered by nonproton ligands at physiological pH levels and activated by a decrease in extracellular pH below 7.0 [11]. They also have a mechanosensitive role in colon ganglia cells, nerve terminals of the aortic arch and the bladder [16]. ASIC proteins, encoded by four genes (*ACCN1*, *ACCN2*, *ACCN3* and *ACCN4*), form at least six subtypes of channels. Activation of ASICs triggers Na^+^ influx but also exhibits calcium permeability, acquiring important functions in learning and memory [17].

### 2.2. Transient Receptor Potential Channel Family

Transient receptor potential (TRP) channels are nonselective cation channels that play an important role in Ca^2+^ signaling. TRPs act as sensors of light, touch or mechanical pain [18], leading to membrane depolarization through Ca^2+^ influx. They form tetrameric cation-selective complexes of either identical or distinct subunits, each with six transmembrane domains with both the N- and C-termini in the intracellular compartment [19] (Figure 1B).

In mammals, the TRP family is composed of 28 members distributed in six subfamilies [19,20]: TRPC (canonical channels), TRPV (vanilloid), TRPA (ankyrin), TRPM (melastatin), TRPML (mucolipin) and TRPP (polycystin). The TRPC, TRPV and TRPA subfamilies contain ankyrin repeat motifs present in tandem copies that confer elastic properties, playing a key role in mechanosensing [21]. Most TRPs are expressed in the plasmatic membrane, but some of them also exert their function at intracellular membranes, playing a role in endolysosomal system regulation [22,23].

TRPC members play important roles in different tissues, including the cardiovascular system, skeletal muscles, lung, kidneys, salivary glands, reproductive system, immune system, and nervous system, as reviewed in [24,25].

In mammals, a sole member (TRPA1) composes the TRPA subfamily. The expression of this channel has been described in several cell types, such as sensory neurons, epithelial cells, melanocytes and keratinocytes, and it can be activated not only by mechanical cues but also by pH changes, thermal changes and different ligands, such as cholesterol or nicotine [26].

Finally, inside the TRPV subfamily, TRPV1–4 are nonselective cation channels that are modestly permeable to Ca^2+^, while TRPV5 and TRPV6 are highly selective Ca^2+^ channels. All of them are widely expressed in mammals, such as neural tissue, kidneys, liver, immune system, heart, smooth muscle, skin, lung and placenta [27].

### 2.3. Two-Pore-Domain Potassium Channel Family

The two-pore-domain potassium channels (K2P) are a diverse family of K^+^ selective channels encoded by fifteen different genes [28] and regulated by mechanical cues as well as other stimuli such as anesthetics and antidepressant agents [29,30], neurotransmitters, posttranslational modifications or temperature [31]. K2Ps are classified into six different subfamilies (TWIK, TREK, TASK, TALK, THIK and TRESK) according to their sequence similarity and functional characteristics [32]. Plasma membrane tension, complemented by other mechanical factors such as protein–protein interactions and cytoskeletal modulation, directly regulates TREK subfamily members [32,33].

Their structure differs from other K^+^ channels, as each K2P subunit contains four transmembrane domains (M1–M4 domains) and two pore-forming domains (P domains), with the N- and C-terminal sides at the cytoplasmic side (Figure 1C). They act as homodimers, as K2P needs four P domains to constitute the K^+^ selective filter [32,34].

K2P channels have a key role in the nervous system, heart and muscles by controlling membrane resting potential and excitability [35,36]. However, they exert important functions in non-excitable tissues, such as the pancreas, by controlling glucagon release [37], surfactant integrity in the lungs [38], and the proliferation and cytolytic functions of natural killer cells [39].

### 2.4. PIEZO Channel Family

The PIEZO family comprises mechanoreceptors Piezo1 and Piezo2, also known as FAM38A and FAM38B, respectively. Piezo1 was first identified and characterized in a mouse neuroblastoma cell line by Coste and colleagues [40], and they later identified Piezo2 by sequence homology. They are both nonselective Ca^2+^ channels that are highly conserved across species and expressed in most tissues of organisms, giving an idea of their biological importance.

PIEZO proteins have an unusually large size compared to other ion channels. They have an overall size of over 2500 amino acids, with a large number of transmembrane regions [40]. Mouse Piezo1 and Piezo2 channels have been structurally characterized by cryoEM [41,42]. Both channels have a similar structure, comprising a homotrimer forming a three-blade propeller plus an extracellular cap [41,42] (Figure 1D).

It is important to note that, while most of the mechanosensitive channels described above exhibit multiple activation mechanisms, the only channels that are principally activated by mechanical stimuli are the PIEZO channels [43,44]. However, it has been shown that although their main activation cue is mechanical stimuli, PIEZO channels can also be modulated by voltage [45].

## 3. Mechanoreceptors in Cancer

It is now known how crucial the mechanical properties of tissues are in both health and disease. Indeed, the mechanical environment of tumors is different from that of healthy tissue [49]. Solid tumors become more rigid, mainly due to an increase in the structural elements of the tumor, such as the number of cancer cells, stromal cells, and components of the ECM, all of which lead to stiffening of the tissue [50]. Furthermore, this characteristic helps the tumor displace the surrounding healthy tissue, allowing it to enlarge and infiltrate the organ [51].

The mechanical environment of tumors can affect their aggressiveness and organotropism. High tumor stiffness is closely related to tumor progression and can drive invasion by modulating the CNN1/β-catenin/N-cadherin pathway, contributing to the binding of cancer cells to blood vessels [52]. There are several cancers that can metastasize to other organs with different mechanical properties, such as breast [53,54], lung [55,56] and prostate cancer [57,58], but it has been shown that the tropism can depend on the mechanical properties of the cancer cells themselves [59].

There are a myriad of mechanisms and molecules involved in the whole process of mechanosensation and tumor progression [60]. However, mechanosensitive ion channels are pivotal players in these processes [2,61]. Here, we review the role of PIEZO channels and some TRPs in cancer, as they are the two most distinctive mechanosensitive ion channel families.

### 3.1. TRPM7

TRPM7 is a TRP mechanosensitive ion channel that mediates the transport of Ca^2+^ and other divalent cations (i.e., Zn^2+^ and Mg^2+^) through the plasma membrane, and it is able to autophosphorylate and phosphorylate different protein substrates through its C-terminal serine/threonine kinase domain [62].

The expression of TRPM7 is altered in several cancers (Table 1). High levels of TRPM7 are correlated with upregulation of the epithelial-mesenchymal transition (EMT) pathway and poor disease-free and overall survival in ovarian cancer cells [63]. Liu and colleagues also described that TRPM7 inhibition reduces intracellular Ca^2+^ levels and attenuates EMT, migration and invasion via PI3K/Akt inhibition [63], and its silencing impairs glycolysis and proliferation [64], suggesting TRPM7 as a valuable marker for ovarian metastasis and a possible therapeutic marker, as well as having been described as a potential prognostic marker for this cancer type [65,66].

In breast cancer, TRPM7 is associated with poor prognosis, cell migration and metastasis [67,68], and its high methylation profile is associated with a good prognosis [69]. However, its role in cell proliferation is not completely clear, as it seems to highly contribute to the proliferation of the adenocarcinoma cell line MCF7 [70] but not the triple-negative breast cancer cell line MDA-MB-231 [71].

TRPM7 is also highly expressed in bladder cancer tissues and cell lines [72], and it is associated with poor clinical outcomes because it is involved in cell proliferation, migration and invasion [72]. The key role of this channel in bladder cancer malignancy can be reverted by its downregulation, which leads to mitochondrial-dependent apoptosis via ERK1/2 signaling and a reduction in metastasis markers such as phospho-FAK and MMP2/9 [73]. The downregulation of TRPM7 can lead to phosphorylation of ERK1/2, promoting the induction of mitochondrial Cytochrome C release or caspase-8 activation, as well as autophagic vacuolization and permanent cell cycle arrest [74]. Other pathways regulated by TRPM7 in bladder cancer cells are the Src, Akt and JNK pathways [75].

In cervical cancer cells, TRPM7 mediates necrotic cell death dependent on acidic pH. As the vagina has an acidic physiological pH, TRPM7 expression activates necrotic death pathways, leading to an inflammatory response in the surrounding tissue. However, after progesterone treatment, TRPM7 expression and activity are inhibited, preventing cervical cancer cell growth and switching cell death from acidotoxic necrosis to apoptosis [76]. There are two microRNAs (miRNAs) able to downregulate TRPM7 in cervical cancer. Both miRNAs, miR-543 [77] and miR-192-5p [78], directly target TRPM7, inhibiting tumor growth, migration and invasion driven by this channel in cervical cancer.

Additionally, TRPM7 also seems to play an essential role in glioblastoma. This channel is highly expressed in human glioblastoma tissues and controls proliferation, migration and invasion in glioblastoma cells [79,80]. TRPM7 activates the JAK2/STAT3 signaling pathway leading to glioma aggravation [79,81], as there is crosstalk between this pathway and ALDH1 and CD133, two glioma stemness markers, suggesting a role of TRPM7 in glioma stem cells [81]. In neuroblastoma, another CNS tumor, TRPM7 promotes a stem-like phenotype and promotes metastasis [82]. Additionally, the evidence suggests that this mechanoreceptor contributes to matrix protein remodeling in neuroblastoma [83].

In prostate cancer, it has been described that TRPM7 expression is much higher in metastatic prostate cancer than in benign prostatic hyperplasia [84]. An increase in Ca^2+^ via TRPM7 drives cell proliferation and migration via Akt and ERK phosphorylation [85]. Under hypoxia, the TRPM7-HIF1α-Annexin A1 signaling axis is essential for the EMT, cell migration and invasion of prostate cancer cells [86]. In this line, TGFβ stimulates the expression of TRPM7, further enhancing EMT in prostate cancer cells [87].

It has been demonstrated that TRPM7 plays a role in other cancers, including gastrointestinal [88,89,90], lung [91], leukemia [92], head and neck carcinoma [93] and neuroblastoma [82,83]. In most cases, TRPM7 leads to the regulation of the PI3K/Akt [63,75,85,94] and MAPK signaling pathways [73,85,94], promoting cell growth, migration and/or invasion.

**Table 1:** Clinical importance and functional analysis of TRPM7 in different tumors.

Cancer Type	Expression	Function	Clinical Features	Molecular Mechanism	References
Ovarian	Upregulated	Proliferation, migration, invasion.	Poor disease-free survival and poor overall survival.	EMT factors upregulation. PI3K/Akt signaling activation. Indirect HIF-1α regulation.	[63,64,65,66]
Breast	Upregulated	Proliferation, migration, invasion.	Poor prognosis.	Proliferation mechanisms are unclear. High *TRPM7* promoter methylation is a good prognostic marker in the luminal A subtype.	[67,68,69,70,71]
Bladder	Upregulated	Proliferation, migration, invasion.	Poor clinical outcomes.	Proliferation mechanisms are unclear. Pro-apoptotic ERK1/2 pathway activation and metastasis markers are downregulated in TRPM7 KD. Src, Akt and JNK upregulation.	[69,70,71]
Cervical	Undetermined	Proliferation, migration, invasion.	Undetermined.	Necrotic cell death regulation miR-543 and miR-192-5p target TRPM7 directly.	[76,77,78]
Glioblastoma	Upregulated	Proliferation, migration, invasion.	Tumor aggravation.	JAK2/STAT3 and Notch1 signaling activation. Glioma stem marker expression.	[79,80,81]
Prostate	Upregulated	Tumorigeneses, migration, invasion.	Undetermined.	Activation of the Akt and ERK signaling pathways. TRPM7-HIF1α-Annexin A1 axis activation.	[84,85,86,94,95]

### 3.2. TRPV4

TRPV4 belongs to the TRPV family and is a nonselective Ca^2+^, Mg^2+^ and Na^+^ permeable channel. Its structure is quite similar to that of other TRP channels, with a homotetramer with a variety of functional domains [96]. Similar to other mechanosensitive channels, TRPV4 has been reported to be involved in different types of cancer.

In breast cancer, TRPV4 activation decreases the viability of two basal breast cancer cell lines with endogenous overexpression of TRPV4. Upon activation, TRPV4 leads to cell death through apoptosis (PARP-1 cleavage) and oncosis (ATP decrease), and this oncosuppressor mechanism has also been shown in vivo [97]. In contrast, TRPV4 was found to be indispensable for breast cancer cell invasion and migration, as its overexpression promoted vesicle formation and actin reorganization in breast cancer tumor cells [98].

TRPV4 is also overexpressed in hepatocellular carcinoma tissues compared to non-tumorous liver tissues. Indeed, Fang and colleagues described that TRPV4 inhibition in vitro depleted cell proliferation, reduced EMT, and led to apoptosis via downregulation of p-ERK while also suppressing tumor growth in xenograft models in vivo [99].

Likewise, TRPV4 is upregulated in colon cancer and associated with a poor prognosis [100]. TRPV4 elicits cancer progression through inhibition of the tumor suppressor PTEN and upregulation of Akt-ZEB1 signaling, which modulates EMT and aggressiveness [101], while its inhibition leads to cell cycle arrest and induction of apoptosis/autophagy [100].

Increased lymph node metastasis, deeper tumor invasion, a higher TNM stage, poor overall survival and poor disease-free survival have been linked to TRPV4 overexpression [102]. Patients with gastric cancer who have hypercalcemia typically have a poor prognosis. Xie and colleagues showed that calcium-sensitive receptor (CasR) activation triggered TRPV4-mediated Ca^2+^ entry, followed by Akt and β-catenin phosphorylation and tumor progression [103]. It has been demonstrated that activating VPAC1 by VIP significantly increased Ca^2+^ entry through TRPV4, triggering several migration and invasion signaling cascades and, in turn, promoting the expression and secretion of VIP [104].

Thus, TRPV4 plays a role in several tumor-related mechanisms, such as proliferation, apoptosis, angiogenesis, migration and invasion. Due to the diverse functions in which TRPV4 intervenes in cancer progression as well as its overexpression compared to healthy tissue, TRPV4 could be a potential pharmacological target with therapeutic benefits in different types of cancer.

### 3.3. Piezo1

Piezo1 belongs to the family of PIEZO channels. It has a homotrimeric structure, where the blades detect cell membrane deformation and generate a force that is transmitted to the intracellular beams, opening the cap and allowing the influx of Ca^2+^ [41].

Since Piezo1 was discovered and characterized, it has been described as playing a role in a myriad of cancers. For instance, malignant breast cancer cell lines had a more aggressive behavior in response to compressive stress, and this phenotype is related to Piezo1 function [105]. Piezo1 acts as a critical sensor of compression stress in these cells, increasing migration and invasion of breast cancer cells by modulating the invadopodium formation and degradation of ECM through metalloproteinases [106] by actin protrusion formation via Src signaling [107]. On the other hand, it has also been shown that Piezo1 activation can attenuate the blebbing mechanism in breast cancer cells, this type of migration is observed in some other cancers [108].

In glioma, Chen and colleagues have shown that Piezo1 can trigger glioma aggressiveness in *D. melanogaster* [109]. Furthermore, Piezo1 overexpression in glioblastoma is correlated with the degree of peritumoral brain edema, a glioblastoma-associated condition that aggravates the patient’s symptoms [110]. Likewise, it seems that Piezo1 expression is positively correlated with ECM organization, cell adhesion, angiogenesis, cell migration and proliferation, making it a predictive marker of poor prognosis [111].

Similarly, Piezo1 is also overexpressed in prostate cancer cells compared to normal tissue, and its upregulation leads to Akt/mTOR activation, promoting cell cycle progression and, subsequently, prostate tumor growth [112]. Shear stress enhances Piezo1 activity in prostatic cancer cells, leading to Src/YAP signaling activation and prostate tumor progression [113].

Piezo1 is also highly expressed in gastric cancer, especially when there is peritoneal metastasis [114]. Piezo1 directly interacts with the trefoil factor family 1 (TFF1) protein, enhancing gastric cancer cell migration in vitro [115]. Loss-of-function assays shed some light on the molecular mechanisms in gastric cancer in which Piezo1 is involved. Piezo1 defects significantly inhibit the oncogenic behavior of gastric cancer cells by downregulating cell proliferation, migration and invasion and enhancing drug sensitivity. Its oncogenic mechanisms may be related to direct activation of the Rho family GTPase members RhoA and Rac1 [116]. Furthermore, Piezo1 upregulates HIF-1α, inducing cell migration via calpain1/2, whereas Piezo1 knockdown inhibits tumor growth and blocks both EMT and angiogenesis in peritoneal metastatic gastric cancer in vivo [114].

In liver cancer, Piezo1 is directly correlated with poor clinical outcomes. Piezo1 recruits Rab5c, a small GTPase, promoting hepatocellular carcinoma via TGF-β signaling [117]. Additionally, Piezo1 is necessary for MAPK and YAP activation and translocation in hepatocellular carcinoma through an independent Hippo signaling mechanism [118]. Recently, Li and colleagues observed that increased matrix stiffness significantly upregulated Piezo1 expression, suppressing HIF-1α ubiquitination, and promoting angiogenesis [119].

However, there are many other cancer types in which Piezo1 is usually upregulated. For instance, Piezo1 upregulation has been shown to trigger ovarian cancer metastasis via Hippo/YAP signaling [120]. Piezo1 mRNA is overexpressed in bladder cancer [121] and colorectal cancer [122], promoting cell growth and invasion. High expression of Piezo1 in pancreatic cancer is associated with poor disease-free survival, and a noninvasive treatment strategy based on ultrasound stimulation of microbubbles induces mitochondrial dysfunction and apoptosis via Piezo1 [123]. However, in contrast to all the other cancer types described, in non-small cell lung cancer patients, Piezo1 is expressed at low levels, and this low expression is linked to poor overall survival due to an increase in cell migration and tumor growth [124].

Thus, Piezo1 seems to be mostly protumoral, acting through diverse mechanisms (Table 2) and playing a key role in certain hallmarks of cancer: migration, invasion and angiogenesis.

### 3.4. Piezo2

Piezo2 is the other member of the PIEZO family, alongside Piezo1, and their structures are similar [42]. Piezo2 also senses mechanical forces on cell membranes, leading to an influx of Ca^2+^. Among its physiological functions, Piezo2 has an excitatory effect in neurons, depolarizing the membrane and causing action potential firing [125]. However, the involvement of Piezo2 in different types of cancer has not yet been widely described.

It has been recently reported that knockout of Piezo2 in SOX2+ medulloblastoma cells reduces local tissue stiffness, improves drug delivery across the blood-tumor barrier, and increases survival by altering WNT/β-catenin signaling between tumor and endothelial cells [126]. Likewise, Piezo2 was found to be upregulated in a glioma xenograft model, and its depletion reduced glioma angiogenesis [127].

In lung cancer, similar to its homolog Piezo1, Piezo2 is negatively correlated with overall survival in non-small cell lung cancer patients [128]. Interestingly, Piezo2 expression is also downregulated in breast cancer cell lines and patient tissue, and it negatively correlates with breast cancer progression. Lou and colleagues found five onco-miRNAs that target *PIEZO2*, which caused a decrease in CDON expression and activation of the Hedgehog pathway [129]. In contrast, Piezo2 is necessary for breast cancer cells to metastasize to the brain, as in the case of MDA-MB-231-BrM2 cells, because it acts as an upstream regulator of the RhoA-mDia pathway associated with invadosome functions [130]. Additionally, Piezo2 is overexpressed in triple-negative breast cancer, and its high expression seems to be correlated with a worse prognosis [131].

Last year, an extensive in silico analysis of Piezo2 expression, genetic relationships with immunological markers, and predictive functions in pan-cancer was performed. This study indicated that Piezo2 expression is cell- and tissue-dependent in different cancer types and is related to prognosis in several tumors [132].

Thus, despite its potential role in multiple cancer types, more research is required to fully understand the roles played by Piezo2 in tumors and the underlying mechanisms through which this mechanosensitive ion influences cancer.

## 4. Mechanosensation of the Immune System and Immune Response to Cancer

It is now clear that mechanosensitive ion channels are essential for physiological tissue functions. Indeed, the immune system is one of the most mechanosensitive tissues in all organisms, as immune cells encounter a wide range of environmental biophysical conditions depending on the tissue in which they are located [133]. Mechanical stimuli acting on immune cells are generated by both the hemodynamic forces and the ECM composition of tissues and can be classified as follows: (i) mechanical stretch by shape changes during cell passage through narrow capillary segments, (ii) shear stress acting on circulating or adherent immune cells such as blood flow, and (iii) changes in the ECM stiffness induced by inflammation.

The immune system response to cancer has proven to be of extreme importance and is indeed one of the most promising arms of treatment in the current panorama, with the development of immunotherapy for cancer treatment. Moreover, pathological conditions such as infection or cancer cause a change in the stiffness of tissues [134,135], affecting the immune response. In recent years, it has been shown that mechanosensitive ion channels play essential roles in the immune system and that they are important for proliferation [136], activation [136,137], migration [138] and cytokine production [137] of immune cells.

### 4.1. TRPA1

TRPA1 is the only member of the TPRA family in mammals and is a nonselective cation channel permeable to Ca^2+^, Na^+^ and K^+^. Similar to other TRP channels, it is a homotetramer [139] and can be activated not only by mechanical stimuli but also by chemical [140,141] and thermal [142,143] cues. Additionally, different agents associated with an inflammatory context seem to modulate TRPA1, such as bacterial lipopolysaccharide [144], reactive oxygen species (ROS) or nitric oxide [145], among others.

TRPA1 expression has been detected in a wide range of immune cells. Some studies have linked the activation of TRPA1 in neutrophils to arthritis [146]. TRPA1 has also been detected in mast cells, where it could play a role in degranulation [147] and anaphylaxis [148], although it is not the only key player, as there are also other TRPA1-independent mechanisms involved.

In macrophages, TRPA1 activation leads to an anti-inflammatory effect [149]. According to Wang et al., this effect is due to the fact that TRPA1 promotes the polarization of macrophages toward the M2 phenotype through an epigenetic mechanism that promotes H3K27 tri-methylation [150].

As the Ca^2+^ response is critical for T-cell activation and cell fate [150,151], TRPA1 plays a key role in the biology of these cells. In a melanoma orthotopic mouse model, *Trpa1^−/−^* mice showed higher CD8+ T-cell activation and tumor infiltration as well as lower tumor progression than *Trpa1^+/+^* animals [152], indicating that TRPA1 diminishes CD8+ T-cell cytotoxicity. Likewise, a recent publication highlights that *Trpa1^−/−^* mice show a lower CD4+/CD8+ T-cell ratio and higher CD8+ cell activation than WT mice, and this TRPA1 deficiency also reduced the percentage of peripheral blood CD19+ B cells [153]. On the other hand, the role of TRPA1 in CD4+ T-cell biology is controversial. While some studies report that TRPA1 restrains CD4+ T-cell activation by a crosstalk mechanism that implies the inhibition of TRPV1 [154], others indicate that its inhibition is correlated with the reduction of CD4+ T-cell activation markers such as CD69 [137,153], thus requiring further research to fully understand the role of TRPA1 in CD4+ T-cell biology.

### 4.2. TRPV4

Apart from having key roles in cancer cells, TRPV4 is also involved in the immune response, with special implications for innate immune cell function.

Some studies have reported that this channel plays a key role in neutrophil responses to proinflammatory stimuli such as migration, adhesion or ROS production [138]. In this regard, genetic and pharmacological inhibition of TRPV4 results in marked protection from acute lung injury [155], as pulmonary function is affected by neutrophil activation and infiltration into the injured lung.

In macrophages, TRPV4 plays a dual role, mediating both proinflammatory (phagocytosis, adhesion and ROS production) [156] and anti-inflammatory (proresolution cytokines and bacterial clearance) functions [157]. In addition, TRPV4 activation has been shown to result in nuclear translocation of YAP/TAZ, a transcriptional coactivator that regulates macrophage polarization toward a proinflammatory phenotype in response to substrate stiffness [158]. Likewise, in a matrix stiffness-dependent manner, TRPV4 also modulates macrophage phagocytosis, promoting a switch in the MAPK signaling pathway [159]. Thus, it should be borne in mind that the therapeutic target of TRPV4 could have effects not only on the cancer cells themselves but also on the innate response against the tumor.

### 4.3. Piezo1

Piezo1 is involved in multiple functions of innate and adaptive immune cells, mainly in the activation of immune cells. For instance, the activation of Piezo1 modulates the polarization of macrophages to an M1 phenotype, and it is involved in the sensing of substrate stiffness, bacterial clearance and proinflammatory cytokine production [136]. According to Geng and colleagues, Piezo1 interacts with TLR4 to coax macrophages to achieve the functions necessary for host defense by remodeling F-actin organization and augmenting phagocytosis [160], proving that Piezo1 can play a role in immunity by interacting with other canonical receptors such as TLR4. In the context of macrophage polarization, Piezo1 silencing in macrophages has been associated with increased expression of different integrins, such as CD11b (αM), β1, β2 and β3 [161], all of which are involved in polarization toward the M2 phenotype. This suggests that Piezo1 can promote macrophage-driven inflammation not only directly but also indirectly by promoting the downregulation of certain integrins needed for M2 polarization, thus inhibiting the pro-healing M2 phenotype.

Dendritic cells are also highly mechanosensitive. Environmental stiffness affects their metabolism and function, as dendritic cells show significantly higher proliferation, activation, cytokine production and upregulation of glucose metabolism on stiff substrates compared to physiological resting stiffness. Although the molecular mechanism underlying this sensing is not yet fully clear, it has been proposed that Piezo1 is one of the key mechanosensors responsible for these processes [162].

Piezo1 is also relevant for the adaptive response, especially for T-cells. Recent studies have shown that Piezo1 plays a major role in T-cell activation, as stretching during immune synapse formation triggers the activation of this channel, leading to Ca^2+^ influx, which in turn activates calpain and results in cytoskeletal rearrangement to optimize TCR signaling [163]. Additionally, some studies have shown that Piezo1 is involved in T-cell differentiation as well. According to Jairaman and colleagues, activation of Piezo1 in CD4+ T-cells selectively restrains T_reg_ cell generation without affecting effector T_h1_ and T_h17_ cell polarization [164]. In this line, it has also been reported that Piezo1 signaling in dendritic cells during antigen presentation activates the production of the proinflammatory cytokine IL-12 and promotes T_h1_ polarization, inhibiting the T_reg_ lineage [165].

All these studies suggest a proinflammatory role for Piezo1. Nevertheless, a recent investigation has shown that targeting Piezo1 confers protection against pancreatic ductal carcinoma (PDAC) and multi-microbial sepsis, as this channel is essential for myeloid-derived suppressor cell (MDSC) infiltration and immunosuppressive functions [166]. Thus, it seems that the role of Piezo1 in the immune system is variable and depends on the physiological and pathological context, illustrating the need for further research to fully understand its precise role in the immune response.

## 5. Discussion

Through this review, we have observed the importance of mechanosensation in both physiology and pathology. In addition to adhesion molecules such as integrins and selectins or specialized surface proteins such as CD197, mechanosensitive ion channels have an essential role in detecting and responding to mechanical cues and adapting to their environment.

**Figure 2:**
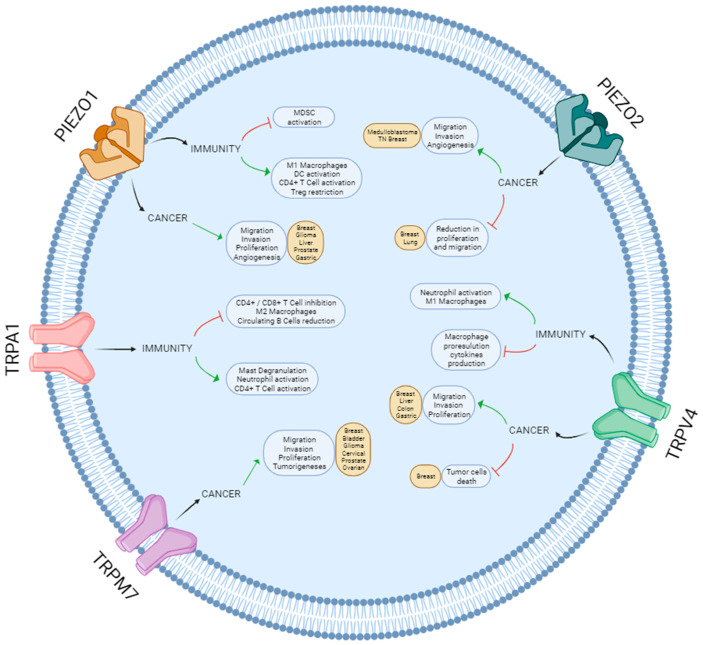
Schematic overview of the roles of Piezo and TRP channels in cancer and immunity.

Most pathological processes are associated with significant changes in the mechanical properties of tissues. One of the most dramatic is cancer, when, as a consequence of excessive cell proliferation and extracellular matrix remodeling, tissue stiffness greatly increases [135,167]. Therefore, mechanosensation is essential in the onset and development of multiple types of tumors. The upregulation of mechanosensitive ion channels is commonly associated with increased proliferation, migration and invasion of tumor cells, which postulates these channels as potential therapeutic targets in these cancers. Nevertheless, a pro-tumoral or anti-tumoral effect of a specific channel depends on the type of cancer [97,108,128] (Figure 2), and the molecular mechanism underlying most of these processes still needs further investigation.

The anti-tumoral immune response is one of the most relevant cancer-related investigations in the current panorama. The balance between the immune response against cancer cells and the immune-evasion mechanisms of the tumor is critical for cancer development, and current work is focused on modulating these processes as a therapeutic approach. Immune cells are constantly subjected to mechanical stimuli, which is critical to their function as they are highly mechanosensitive cells [168]. Indeed, we have observed that mechanosensitive ion channels, particularly the TRP and PIEZO families, are especially important in regulating the immune response. The activation of these channels in immune cells can trigger a plethora of responses leading to a pro- or anti-inflammatory phenotype depending on the cell type, the channel, the stimuli and the environmental conditions.

Targeting mechanosensitive ion channels could thus have a great impact in the clinic, as they play a role in both cancer cells and the immune system. How the different mechanoreceptors can trigger or regulate the immune response against cancer is still not fully understood. Some studies associate the inhibition of these channels with an increase in the anti-tumor response. According to Aykut and colleagues, inhibition of Piezo1 unleashes innate immunity against pancreatic ductal carcinoma since Piezo1 is essential for MSDC immunosuppressive functions [166]. In this line, another study reports that mice with TRPA1 ubiquitous deletion show an increased cytotoxic lymphocyte response against melanoma cells and, consequently, lower tumor progression [152]. However, other studies have concluded that it is the activation of mechanosensitive ion channels that enhances the immune response against cancer. For instance, it has been observed that genetic ablation of Piezo1 in dendritic cells inhibits the generation of T_h1_ cells and drives the development of T_reg_ cells, promoting cancer growth in mice [165]. All this taken together proves the potential of mechanoreceptors for targeting and modulating the immune system not only for cancer but for all pathological situations as well.

## 6. Challenges and Future Perspectives

Despite the great advances in the past few years in understanding the role of mechanosensitive ion channels in cancer and immunity, this field is still in its infancy. Although the different families of mechanoreceptors may share some common hallmarks, they are also very different among them, and thinking of mechanosensation as a global cue in cancer may lead to a mistake. Furthermore, there can be possible compensating mechanisms when one of the receptors sensing is disturbed, which has not been studied in the literature yet.

When researching mechanoreceptors in vitro, one of the main challenges encountered can be the limitations of 2D cell culturing. Most experiments are performed in this canonical way, and their results are widely accepted. However, we must bear in mind that 2D culturing adds an extra and uncontrollable variable: the spontaneous activation of different mechanosensitive ion channels and the lack of the physiological mechanical characteristics of the tissue studied. That is why optimizing 3D cultures with similar mechanical properties to the physiological tissue for the study of mechanoreceptors would be ideal. Organoids are an excellent example of 3D culture. Indeed, cancer-derived organoids have proven to be very useful in the field, as they are a better model than animals and allow the study of the heterogenicity of the tumors while maintaining the mutational profile and the phenotypic characteristics of the original patient. Moreover, these cultures represent a much better model to investigate the biophysics of the tumor and the contribution of mechanoreceptors compared to the classical 2D cell culture. However, the use of organoids still faces important challenges, such as high intra- and intertumoral variability depending on the biopsy recession and the culturing methods.

In conclusion, mechanosensitive ion channels can influence a myriad of different cellular pathways, depending on the cell context. As such, their role in cancer and immunity is complex, changing with the type of stimulus, the cell lineage, the interaction with other proteins, and much more. There is still a gap in knowledge regarding the existence and function of splice variants, unknown mutations, heterotetramer configuration, and crosstalk between the different mechanoreceptors. However, their potential to be therapeutically exploited in cancer is undeniable and promising, and it has turned mechanoreceptors into a hot topic molecule not only in cancer research but also in all fields of biology.

## Figures and Tables

**Table 2:** Clinical importance and functional analysis of Piezo1 in different cancer types.

Cancer Type	Expression	Function	Clinical Features	Molecular Mechanism	References
Breast	Upregulated	Migration, invasion.	Poor overall survival.	Mechanical stress sensor. Actin protrusion formation via Src. Bleb-driven cell migration attenuation.	[101,102,103,104]
Glioma	Upregulated	Proliferation, migration, invasion, angiogenesis.	Glioblastoma aggravation, poor prognosis.	ECM remodeling.	[105,106,107]
Prostate	Upregulated	Proliferation, migration, invasion.	Poor overall survival.	Akt/mTOR signaling activation. Src/YAP signaling activation.	[108,109]
Gastric	Upregulated	Proliferation, migration, invasion, angiogenesis.	Poor overall survival.	TFF1 inhibition. RhoA and Rac1 activation Calpain1/2 activation via HIF-1α upregulation.	[110,111,112]
Liver	Upregulated	Migration, invasion, angiogenesis.	Poor clinical outcomes.	TGF-β activation via Rac5c. YAP activation in a Hippo-independent signaling mechanism. Mechanical stress sensor.	[113,114]

## Data Availability

Not applicable.

## References

[1] Rømer A.M.A., Thorseth M.-L., Madsen D.H. (2021). Immune Modulatory Properties of Collagen in Cancer. Front. Immunol..

[2] Basson M.D., Zeng B., Downey C., Sirivelu M.P., Tepe J.J. (2014). Increased extracellular pressure stimulates tumor proliferation by a mechanosensitive calcium channel and PKC-β. Mol. Oncol..

[3] Kudou M., Shiozaki A., Yamazato Y., Katsurahara K., Kosuga T., Shoda K., Arita T., Konishi H., Komatsu S., Kubota T. (2019). The expression and role of TRPV2 in esophageal squamous cell carcinoma. Sci. Rep..

[4] Canales Coutiño B., Mayor R. (2021). Mechanosensitive ion channels in cell migration. Cells Dev..

[5] Martino F., Perestrelo A.R., Vinarsky V., Pagliari S., Forte G. (2018). Cellular Mechanotransduction: From Tension to Function. Front. Physiol..

[6] Gu Y., Gu C. (2014). Physiological and Pathological Functions of Mechanosensitive Ion Channels. Mol. Neurobiol..

[7] Árnadóttir J., Chalfie M. (2010). Eukaryotic Mechanosensitive Channels. Annu. Rev. Biophys..

[8] Prevarskaya N., Skryma R., Shuba Y. (2018). Ion Channels in Cancer: Are Cancer Hallmarks Oncochannelopathies?. Physiol. Rev..

[9] Kellenberger S., Schild L. (2002). Epithelial Sodium Channel/Degenerin Family of Ion Channels: A Variety of Functions for a Shared Structure. Physiol. Rev..

[10] Hanukoglu I., Hanukoglu A. (2016). Epithelial sodium channel (ENaC) family: Phylogeny, structure–function, tissue distribution, and associated inherited diseases. Gene.

[11] Cheng Y.-R., Jiang B.-Y., Chen C.-C. (2018). Acid-sensing ion channels: Dual function proteins for chemo-sensing and mechano-sensing. J. Biomed. Sci..

[12] Hanukoglu I. (2017). ASIC and ENaC type sodium channels: Conformational states and the structures of the ion selectivity filters. FEBS J..

[13] Christensen B.M., Perrier R., Wang Q., Zuber A.M., Maillard M., Mordasini D., Malsure S., Ronzaud C., Stehle J.-C., Rossier B.C. (2010). Sodium and Potassium Balance Depends on αENaC Expression in Connecting Tubule. J. Am. Soc. Nephrol..

[14] Bhalla V., Hallows K.R. (2008). Mechanisms of ENaC Regulation and Clinical Implications. J. Am. Soc. Nephrol..

[15] Matalon S., Bartoszewski R., Collawn J.F., Hakansson A.P., Orihuela C.J., Bogaert D., Terryah S.T., Fellner R.C., Ahmad S., Moore P.J. (2015). Role of epithelial sodium channels in the regulation of lung fluid homeostasis. Am. J. Physiol.-Lung Cell. Mol. Physiol..

[16] Barth D., Fronius M. (2019). Shear force modulates the activity of acid-sensing ion channels at low pH or in the presence of non-proton ligands. Sci. Rep..

[17] Wemmie J.A., Chen J., Askwith C.C., Hruska-Hageman A.M., Price M.P., Nolan B.C., Yoder P.G., Lamani E., Hoshi T., Freeman J.H. (2002). The Acid-Activated Ion Channel ASIC Contributes to Synaptic Plasticity, Learning, and Memory. Neuron.

[18] Eijkelkamp N., Quick K., Wood J.N. (2013). Transient Receptor Potential Channels and Mechanosensation. Annu. Rev. Neurosci..

[19] Venkatachalam K., Montell C. (2007). TRP channels. Annu. Rev. Biochem..

[20] Samanta A., Hughes T.E.T., Moiseenkova-Bell V.Y. (2018). Transient Receptor Potential (TRP) Channels. Subcell. Biochem..

[21] Tang Y.Q., Lee S.A., Rahman M., Vanapalli S.A., Lu H., Schafer W.R. (2020). Ankyrin Is an Intracellular Tether for TMC Mechanotransduction Channels. Neuron.

[22] Dong X.-P., Wang X., Xu H. (2010). TRP channels of intracellular membranes. J. Neurochem..

[23] Chen C.C., Krogsaeter E., Grimm C. (2021). Two-pore and TRP cation channels in endolysosomal osmo-/mechanosensation and volume regulation. Biochim. Biophys. Acta Mol. Cell Res..

[24] Wang H., Cheng X., Tian J., Xiao Y., Tian T., Xu F., Hong X., Zhu M.X. (2020). TRPC channels: Structure, function, regulation and recent advances in small molecular probes. Pharmacol. Ther..

[25] Chen X., Sooch G., Demaree I.S., White F.A., Obukhov A.G. (2020). Transient Receptor Potential Canonical (TRPC) Channels: Then and Now. Cells.

[26] Naert R., López-Requena A., Talavera K. (2021). TRPA1 Expression and Pathophysiology in Immune Cells. Int. J. Mol. Sci..

[27] Wu L.-J., Sweet T.-B., Clapham D.E. (2010). International Union of Basic and Clinical Pharmacology. LXXVI. Current Progress in the Mammalian TRP Ion Channel Family. Pharmacol. Rev..

[28] Feliciangeli S., Chatelain F.C., Bichet D., Lesage F. (2015). The family of K2P channels: Salient structural and functional properties. J. Physiol..

[29] Patel A.J., Honore E. (2003). 2P domain K^+^ channels: Novel pharmacological targets for volatile general anesthetics. Adv. Exp. Med. Biol..

[30] Kennard L.E., Chumbley J.R., Ranatunga K.M., Armstrong S.J., Veale E.L., Mathie A. (2005). Inhibition of the human two-pore domain potassium channel, TREK-1, by fluoxetine and its metabolite norfluoxetine. Br. J. Pharmacol..

[31] Schneider E.R., Anderson E.O., Gracheva E.O., Bagriantsev S.N. (2014). Temperature Sensitivity of Two-Pore (K2P) Potassium Channels. Curr. Top Membr..

[32] Enyedi P., Czirják G. (2010). Molecular Background of Leak K^+^ Currents: Two-Pore Domain Potassium Channels. Physiol. Rev..

[33] Lengyel M., Enyedi P., Czirják G. (2021). Negative Influence by the Force: Mechanically Induced Hyperpolarization via K_2P_ Background Potassium Channels. Int. J. Mol. Sci..

[34] Zúñiga L., Zúñiga R. (2016). Understanding the Cap Structure in K2P Channels. Front. Physiol..

[35] Lotshaw D.P. (2007). Biophysical, pharmacological, and functional characteristics of cloned and native mammalian two-pore domain K^+^ channels. Cell Biochem. Biophys..

[36] Mathie A., Veale E.L. (2007). Therapeutic potential of neuronal two-pore domain potassium-channel modulators. Curr. Opin. Investig. Drugs.

[37] Dadi P.K., Luo B., Vierra N.C., Jacobson D.A. (2015). TASK-1 Potassium Channels Limit Pancreatic α-Cell Calcium Influx and Glucagon Secretion. Mol. Endocrinol..

[38] Schwingshackl A., Lopez B., Teng B., Luellen C., Lesage F., Belperio J., Olcese R., Waters C.M. (2017). Hyperoxia treatment of TREK-1/TREK-2/TRAAK-deficient mice is associated with a reduction in surfactant proteins. Am. J. Physiol. Lung Cell. Mol. Physiol..

[39] Schulte-Mecklenbeck A., Bittner S., Ehling P., Döring F., Wischmeyer E., Breuer J., Herrmann A.M., Wiendl H., Meuth S.G., Gross C.C. (2015). The two-pore domain K_2_P channel TASK2 drives human NK-cell proliferation and cytolytic function. Eur. J. Immunol..

[40] Coste B., Mathur J., Schmidt M., Earley T.J., Ranade S., Petrus M.J., Dubin A.E., Patapoutian A. (2010). Piezo1 and Piezo2 Are Essential Components of Distinct Mechanically Activated Cation Channels. Science.

[41] Zhao Q., Zhou H., Chi S., Wang Y., Wang J., Geng J., Wu K., Liu W., Zhang T., Dong M.-Q. (2018). Structure and mechanogating mechanism of the Piezo1 channel. Nature.

[42] Wang L., Zhou H., Zhang M., Liu W., Deng T., Zhao Q., Li Y., Lei J., Li X., Xiao B. (2019). Structure and mechanogating of the mammalian tactile channel PIEZO2. Nature.

[43] Zhao Q., Wu K., Geng J., Chi S., Wang Y., Zhi P., Zhang M., Xiao B. (2016). Ion Permeation and Mechanotransduction Mechanisms of Mechanosensitive Piezo Channels. Neuron.

[44] Syeda R., Florendo M.N., Cox C.D., Kefauver J.M., Santos J.S., Martinac B., Patapoutian A. (2016). Piezo1 Channels Are Inherently Mechanosensitive. Cell Rep..

[45] Moroni M., Servin-Vences M.R., Fleischer R., Sánchez-Carranza O., Lewin G.R. (2018). Voltage gating of mechanosensitive PIEZO channels. Nat. Commun..

[46] Sun D., Liu S., Li S., Zhang M., Yang F., Wen M., Shi P., Wang T., Pan M., Chang S. (2020). Structural insights into human acid-sensing ion channel 1a inhibition by snake toxin mambalgin1. eLife.

[47] Nadezhdin K.D., Talyzina I.A., Parthasarathy A., Neuberger A., Zhang D.X., Sobolevsky A.I. (2023). Structure of human TRPV4 in complex with GTPase RhoA. Nat. Commun..

[48] Brohawn S.G., del Mármol J., MacKinnon R. (2012). Crystal structure of the human K2P TRAAK, a lipid- and mechano-sensitive K+ ion channel. Science.

[49] Levental K.R., Yu H., Kass L., Lakins J.N., Egeblad M., Erler J.T., Fong S.F.T., Csiszar K., Giaccia A., Weninger W. (2009). Matrix Crosslinking Forces Tumor Progression by Enhancing Integrin Signaling. Cell.

[50] Pickup M.W., Mouw J.K., Weaver V.M. (2014). The extracellular matrix modulates the hallmarks of cancer. EMBO Rep..

[51] Jain R.K., Martin J.D., Stylianopoulos T. (2014). The Role of Mechanical Forces in Tumor Growth and Therapy. Annu. Rev. Biomed. Eng..

[52] Reid S.E., Kay E.J., Neilson L.J., Henze A.T., Serneels J., McGhee E.J., Dhayade S., Nixon C., Mackey J.B., Santi A. (2017). Tumor matrix stiffness promotes metastatic cancer cell interaction with the endothelium. EMBO J..

[53] Kuchuk I., Hutton B., Moretto P., Ng T., Addison C., Clemons M. (2013). Incidence, consequences and treatment of bone metastases in breast cancer patients—Experience from a single cancer centre. J. Bone Oncol..

[54] Weil R.J., Palmieri D.C., Bronder J.L., Stark A.M., Steeg P.S. (2005). Breast Cancer Metastasis to the Central Nervous System. Am. J. Pathol..

[55] Akoury E., Luna A.S.R.G., Ahangar P., Gao X., Zolotarov P., Weber M.H., Rosenzweig D.H. (2019). Anti-Tumor Effects of Low Dose Zoledronate on Lung Cancer-Induced Spine Metastasis. J. Clin. Med..

[56] Barnholtz-Sloan J.S., Sloan A.E., Davis F.G., Vigneau F.D., Lai P., Sawaya R.E. (2004). Incidence Proportions of Brain Metastases in Patients Diagnosed (1973 to 2001) in the Metropolitan Detroit Cancer Surveillance System. J. Clin. Oncol..

[57] Roudier M.P., Corey E., True L.D., Hiagno C.S., Ott S.M., Vessella R.L. (2004). Histological, Immunophenotypic and Histomorphometric Characterization of Prostate Cancer Bone Metastases. Cancer Treat. Res..

[58] Boxley P.J., Smith D.E., Gao D., Kessler E.R., Echalier B., Bernard B., Ormond D.R., Lam E.T., Kavanagh B.D., Flaig T.W. (2021). Prostate Cancer Central Nervous System Metastasis in a Contemporary Cohort. Clin. Genitourin. Cancer.

[59] Tang K., Xin Y., Li K., Chen X., Tan Y. (2021). Cell Cytoskeleton and Stiffness Are Mechanical Indicators of Organotropism in Breast Cancer. Biology.

[60] Li X., Wang J. (2020). Mechanical tumor microenvironment and transduction: Cytoskeleton mediates cancer cell invasion and metastasis. Int. J. Biol. Sci..

[61] Pethő Z., Najder K., Bulk E., Schwab A. (2019). Mechanosensitive ion channels push cancer progression. Cell Calcium.

[62] Yee N.S., Kazi A.A., Yee R.K. (2014). Cellular and Developmental Biology of TRPM7 Channel-Kinase: Implicated Roles in Cancer. Cells.

[63] Liu L., Wu N., Wang Y., Zhang X., Xia B., Tang J., Cai J., Zhao Z., Liao Q., Wang J. (2019). TRPM7 promotes the epithelial-mesenchymal transition in ovarian cancer through the calcium-related PI3K/AKT oncogenic signaling. J. Exp. Clin. Cancer Res..

[64] Chen Y., Liu L., Xia L., Wu N., Wang Y., Li H., Chen X., Zhang X., Liu Z., Zhu M. (2022). TRPM7 silencing modulates glucose metabolic reprogramming to inhibit the growth of ovarian cancer by enhancing AMPK activation to promote HIF-1α degradation. J. Exp. Clin. Cancer Res..

[65] Wang J., Xiao L., Luo C.-H., Zhou H., Hu J., Tang Y.-X., Fang K.-N., Zhang Y. (2014). Overexpression of TRPM7 is Associated with Poor Prognosis in Human Ovarian Carcinoma. Asian Pac. J. Cancer Prev..

[66] Wang J., Liao Q.-J., Zhang Y., Zhou H., Luo C.-H., Tang J., Wang Y., Tang Y., Zhao M., Zhao X.-H. (2014). TRPM7 is required for ovarian cancer cell growth, migration and invasion. Biochem. Biophys. Res. Commun..

[67] Davis F.M., Azimi I., Faville R.A., Peters A.A., Jalink K., Putney J.W., Goodhill G.J., Thompson E.W., Roberts-Thomson S.J., Monteith G.R. (2014). Induction of epithelial–mesenchymal transition (EMT) in breast cancer cells is calcium signal dependent. Oncogene.

[68] Middelbeek J., Kuipers A.J., Henneman L., Visser D., Eidhof I., van Horssen R., Wieringa B., Canisius S.V., Zwart W., Wessels L.F. (2012). TRPM7 Is Required for Breast Tumor Cell Metastasis. Cancer Res.

[69] Wang Y., Lu R., Chen P., Cui R., Ji M., Zhang X., Hou P., Qu Y. (2022). Promoter methylation of transient receptor potential melastatin-related 7 (TRPM7) predicts a better prognosis in patients with Luminal A breast cancers. BMC Cancer.

[70] Guilbert A., Gautier M., Dhennin-Duthille I., Haren N., Sevestre H., Ouadid-Ahidouch H. (2009). Evidence that TRPM7 is required for breast cancer cell proliferation. Am. J. Physiol. Cell Physiol..

[71] Guilbert A., Gautier M., Dhennin-Duthille I., Rybarczyk P., Sahni J., Sevestre H., Scharenberg A.M., Ouadid-Ahidouch H. (2013). Transient receptor potential melastatin 7 is involved in oestrogen receptor-negative metastatic breast cancer cells migration through its kinase domain. Eur. J. Cancer.

[72] Gao S.-L., Kong C.-Z., Zhang Z., Li Z.-L., Bi J.-B., Liu X.-K. (2017). TRPM7 is overexpressed in bladder cancer and promotes proliferation, migration, invasion and tumor growth. Oncol. Rep..

[73] Cao R., Meng Z., Liu T., Wang G., Qian G., Cao T., Guan X., Dan H., Xiao Y., Wang X. (2016). Decreased TRPM7 inhibits activities and induces apoptosis of bladder cancer cells via ERK1/2 pathway. Oncotarget.

[74] Cagnol S., Chambard J.C. (2010). ERK and cell death: Mechanisms of ERK-induced cell death--apoptosis, autophagy and senescence. FEBS J..

[75] Lee E.H., Chun S.Y., Kim B., Yoon B.H., Lee J.N., Kim B.S., Yoo E.S., Lee S., Song P.H., Kwon T.G. (2020). Knockdown of TRPM7 prevents tumor growth, migration, and invasion through the Src, Akt, and JNK pathway in bladder cancer. BMC Urol..

[76] Numata T., Okada Y., Sato-Numata K. (2019). TRPM7 is involved in acid-induced necrotic cell death in a manner sensitive to progesterone in human cervical cancer cells. Physiol. Rep..

[77] Liu X., Gan L., Zhang J. (2019). miR-543 inhibites cervical cancer growth and metastasis by targeting TRPM7. Chem. Biol. Interact..

[78] Dong R.F., Zhuang Y.J., Wang Y., Zhang Z.Y., Xu X.Z., Mao Y.R., Yu J.J. (2021). Tumor suppressor miR-192-5p targets TRPM7 and inhibits proliferation and invasion in cervical cancer. Kaohsiung J. Med. Sci..

[79] Wan J., Guo A.A., King P., Guo S., Saafir T., Jiang Y., Liu M. (2020). TRPM7 Induces Tumorigenesis and Stemness Through Notch Activation in Glioma. Front. Pharmacol..

[80] Leng T.-D., Li M.-H., Shen J.-F., Liu M.-L., Li X.-B., Sun H.-W., Branigan D., Zeng Z., Si H.-F., Li J. (2015). Suppression of TRPM7 Inhibits Proliferation, Migration, and Invasion of Malignant Human Glioma Cells. CNS Neurosci. Ther..

[81] Liu M., Inoue K., Leng T., Guo S., Xiong Z.-G. (2014). TRPM7 channels regulate glioma stem cell through STAT3 and Notch signaling pathways. Cell Signal..

[82] Middelbeek J., Visser D., Henneman L., Kamermans A., Kuipers A.J., Hoogerbrugge P.M., Jalink K., van Leeuwen F.N. (2015). TRPM7 maintains progenitor-like features of neuroblastoma cells: Implications for metastasis formation. Oncotarget.

[83] Visser D., Langeslag M., Kedziora K.M., Klarenbeek J., Kamermans A., Horgen F.D., Fleig A., van Leeuwen F.N., Jalink K. (2013). TRPM7 triggers Ca^2+^ sparks and invadosome formation in neuroblastoma cells. Cell Calcium.

[84] Chen L., Cao R., Wang G., Yuan L., Qian G., Guo Z., Wu C.-L., Wang X., Xiao Y. (2017). Downregulation of TRPM7 suppressed migration and invasion by regulating epithelial–mesenchymal transition in prostate cancer cells. Med. Oncol..

[85] Sun Y., Sukumaran P., Varma A., Derry S., Sahmoun A.E., Singh B.B. (2014). Cholesterol-induced activation of TRPM7 regulates cell proliferation, migration, and viability of human prostate cells. Biochim. Biophys. Acta (BBA)-Mol. Cell Res..

[86] Yang F., Cai J., Zhan H., Situ J., Li W., Mao Y., Luo Y. (2020). Suppression of TRPM7 Inhibited Hypoxia-Induced Migration and Invasion of Androgen-Independent Prostate Cancer Cells by Enhancing RACK1-Mediated Degradation of HIF-1*α*. Oxidative Med. Cell. Longev..

[87] Sun Y., Schaar A., Sukumaran P., Dhasarathy A., Singh B.B. (2018). TGFβ-induced epithelial-to-mesenchymal transition in prostate cancer cells is mediated via TRPM7 expression. Mol. Carcinog..

[88] Pugliese D., Armuzzi A., Castri F., Benvenuto R., Mangoni A., Guidi L., Gasbarrini A., Rapaccini G.L., Wolf F.I., Trapani V. (2020). TRPM7 is overexpressed in human IBD-related and sporadic colorectal cancer and correlates with tumor grade. Dig. Liver Dis..

[89] Kim B.J., Park E.J., Lee J.H., Jeon J.-H., Kim S.J., So I. (2008). Suppression of transient receptor potential melastatin 7 channel induces cell death in gastric cancer. Cancer Sci..

[90] Lefebvre T., Rybarczyk P., Bretaudeau C., Vanlaeys A., Cousin R., Brassart-Pasco S., Chatelain D., Dhennin-Duthille I., Ouadid-Ahidouch H., Brassart B. (2020). TRPM7/RPSA Complex Regulates Pancreatic Cancer Cell Migration. Front. Cell Dev. Biol..

[91] Luanpitpong S., Rodboon N., Samart P., Vinayanuwattikun C., Klamkhlai S., Chanvorachote P., Rojanasakul Y., Issaragrisil S. (2020). A novel TRPM7/O-GlcNAc axis mediates tumour cell motility and metastasis by stabilising c-Myc and caveolin-1 in lung carcinoma. Br. J. Cancer.

[92] Takahashi K., Umebayashi C., Numata T., Honda A., Ichikawa J., Hu Y., Yamaura K., Inoue R. (2018). TRPM7-mediated spontaneous Ca^2+^ entry regulates the proliferation and differentiation of human leukemia cell line K562. Physiol. Rep..

[93] Qiao W., Lan X., Ma H., Chan J., Lui V., Yeung K., Kwong D., Hu Z., Tsoi J., Matinlinna J. (2019). Effects of Salivary Mg on Head and Neck Carcinoma via TRPM7. J. Dent. Res..

[94] Luo Y., Wu J.-Y., Lu M.-H., Shi Z., Na N., Di J.-M. (2016). Carvacrol Alleviates Prostate Cancer Cell Proliferation, Migration, and Invasion through Regulation of PI3K/Akt and MAPK Signaling Pathways. Oxidative Med. Cell. Longev..

[95] Sun Y., Selvaraj S., Varma A., Derry S., Sahmoun A.E., Singh B.B. (2013). Increase in serum Ca^2+^/Mg^2+^ ratio promotes proliferation of prostate cancer cells by activating TRPM7 channels. J. Biol. Chem..

[96] Toft-Bertelsen T.L., MacAulay N. (2021). TRPing to the Point of Clarity: Understanding the Function of the Complex TRPV4 Ion Channel. Cells.

[97] Peters A.A., Jamaludin S.Y.N., Yapa K.T.D.S., Chalmers S., Wiegmans A.P., Lim H.F., Milevskiy M.J.G., Azimi I., Davis F.M., Northwood K.S. (2017). Oncosis and apoptosis induction by activation of an overexpressed ion channel in breast cancer cells. Oncogene.

[98] Lee W.H., Choong L.Y., Mon N.N., Lu S., Lin Q., Pang B., Yan B., Krishna V.S.R., Singh H., Tan T.Z. (2016). TRPV4 Regulates Breast Cancer Cell Extravasation, Stiffness and Actin Cortex. Sci. Rep..

[99] Fang Y., Liu G., Xie C., Qian K., Lei X., Liu Q., Liu G., Cao Z., Fu J., Du H. (2018). Pharmacological inhibition of TRPV4 channel suppresses malignant biological behavior of hepatocellular carcinoma via modulation of ERK signaling pathway. Biomed. Pharmacother..

[100] Liu X., Zhang P., Xie C., Sham K.W.Y., Ng S.S.M., Chen Y., Cheng C.H.K. (2019). Activation of PTEN by inhibition of TRPV4 suppresses colon cancer development. Cell Death Dis..

[101] Zhang P., Xu J., Zhang H., Liu X.-Y. (2021). Identification of TRPV4 as a novel target in invasiveness of colorectal cancer. BMC Cancer.

[102] Wang H., Zhang B., Wang X., Mao J., Li W., Sun Y., Yuan Y., Ben Q., Hua L., Qian A. (2020). TRPV4 Overexpression Promotes Metastasis Through Epithelial–Mesenchymal Transition in Gastric Cancer and Correlates with Poor Prognosis. OncoTargets Ther..

[103] Xie R., Xu J., Xiao Y., Wu J., Wan H., Tang B., Liu J., Fan Y., Wang S., Wu Y. (2017). Calcium Promotes Human Gastric Cancer via a Novel Coupling of Calcium-Sensing Receptor and TRPV4 Channel. Cancer Res.

[104] Tang B., Wu J., Zhu M.X., Sun X., Liu J., Xie R., Dong T.X., Xiao Y., Carethers J.M., Yang S. (2019). VPAC1 couples with TRPV4 channel to promote calcium-dependent gastric cancer progression via a novel autocrine mechanism. Oncogene.

[105] Li C., Rezania S., Kammerer S., Sokolowski A., Devaney T., Gorischek A., Jahn S., Hackl H., Groschner K., Windpassinger C. (2015). Piezo1 forms mechanosensitive ion channels in the human MCF-7 breast cancer cell line. Sci. Rep..

[106] Yu Y., Wu X., Liu S., Zhao H., Li B., Zhao H., Feng X. (2020). Piezo1 regulates migration and invasion of breast cancer cells via modulating cell mechanobiological properties. Acta Biochim. Biophys. Sin..

[107] Luo M., Cai G., Ho K.K.Y., Wen K., Tong Z., Deng L., Liu A.P. (2022). Compression enhances invasive phenotype and matrix degradation of breast cancer cells via Piezo1 activation. BMC Mol. Cell Biol..

[108] O’Callaghan P., Engberg A., Eriksson O., Fatsis-Kavalopoulos N., Stelzl C., Sanchez G., Idevall-Hagren O., Kreuger J. (2022). Piezo1 activation attenuates thrombin-induced blebbing in breast cancer cells. J. Cell Sci..

[109] Chen X., Wanggou S., Bodalia A., Zhu M., Dong W., Fan J.J., Yin W.C., Min H.-K., Hu M., Draghici D. (2018). A Feedforward Mechanism Mediated by Mechanosensitive Ion Channel PIEZO1 and Tissue Mechanics Promotes Glioma Aggression. Neuron.

[110] Qu S., Hu T., Qiu O., Su Y., Gu J., Xia Z. (2020). Effect of Piezo1 Overexpression on Peritumoral Brain Edema in Glioblastomas. Am. J. Neuroradiol..

[111] Zhou W., Liu X., van Wijnbergen J.W.M., Yuan L., Liu Y., Zhang C., Jia W. (2020). Identification of PIEZO1 as a potential prognostic marker in gliomas. Sci. Rep..

[112] Han Y., Liu C., Zhang D., Men H., Huo L., Geng Q., Wang S., Gao Y., Zhang W., Zhang Y. (2019). Mechanosensitive ion channel Piezo1 promotes prostate cancer development through the activation of the Akt/mTOR pathway and acceleration of cell cycle. Int. J. Oncol..

[113] Kim O.-H., Choi Y.W., Park J.H., Hong S.A., Hong M., Chang I.H., Lee H.J. (2022). Fluid shear stress facilitates prostate cancer metastasis through Piezo1-Src-YAP axis. Life Sci..

[114] Wang X., Cheng G., Miao Y., Qiu F., Bai L., Gao Z., Huang Y., Dong L., Niu X., Wang X. (2021). Piezo type mechanosensitive ion channel component 1 facilitates gastric cancer omentum metastasis. J. Cell. Mol. Med..

[115] Yang X.-N., Lu Y.-P., Liu J.-J., Huang J.-K., Liu Y.-P., Xiao C.-X., Jazag A., Ren J.-L., Guleng B. (2014). Piezo1 Is as a Novel Trefoil Factor Family 1 Binding Protein that Promotes Gastric Cancer Cell Mobility In Vitro. Dig. Dis. Sci..

[116] Zhang J., Zhou Y., Huang T., Wu F., Liu L., Kwan J.S.H., Cheng A.S.L., Yu J., To K.F., Kang W. (2018). PIEZO1 functions as a potential oncogene by promoting cell proliferation and migration in gastric carcinogenesis. Mol. Carcinog..

[117] Li Y.-M., Xu C., Sun B., Zhong F.-J., Cao M., Yang L.-Y. (2022). Piezo1 promoted hepatocellular carcinoma progression and EMT through activating TGF-β signaling by recruiting Rab5c. Cancer Cell Int..

[118] Liu S., Xu X., Fang Z., Ning Y., Deng B., Pan X., He Y., Yang Z., Huang K., Li J. (2021). Piezo1 impairs hepatocellular tumor growth via deregulation of the MAPK-mediated YAP signaling pathway. Cell Calcium.

[119] Li M., Zhang X., Wang M., Wang Y., Qian J., Xing X., Wang Z., You Y., Guo K., Chen J. (2022). Activation of Piezo1 contributes to matrix stiffness-induced angiogenesis in hepatocellular carcinoma. Cancer Commun..

[120] Xiong Y., Dong L., Bai Y., Tang H., Li S., Luo D., Liu F., Bai J., Yang S., Song X. (2022). Piezo1 activation facilitates ovarian cancer metastasis via Hippo/YAP signaling axis. Channels.

[121] Etem E., Ceylan G.G., Özaydın S., Ceylan C., Özercan I., Kuloğlu T. (2018). The increased expression of Piezo1 and Piezo2 ion channels in human and mouse bladder carcinoma. Adv. Clin. Exp. Med..

[122] Sun Y., Li M., Liu G., Zhang X., Zhi L., Zhao J., Wang G. (2020). The function of Piezo1 in colon cancer metastasis and its potential regulatory mechanism. J. Cancer Res. Clin. Oncol..

[123] Song Y., Chen J., Zhang C., Xin L., Li Q., Liu Y., Zhang C., Li S., Huang P. (2022). Mechanosensitive channel Piezo1 induces cell apoptosis in pancreatic cancer by ultrasound with microbubbles. iScience.

[124] McHugh B.J., Murdoch A., Haslett C., Sethi T. (2012). Loss of the Integrin-Activating Transmembrane Protein Fam38A (Piezo1) Promotes a Switch to a Reduced Integrin-Dependent Mode of Cell Migration. PLoS ONE.

[125] Szczot M., Nickolls A.R., Lam R.M., Chesler A.T. (2021). The Form and Function of PIEZO2. Annu. Rev. Biochem..

[126] Chen X., Momin A., Wanggou S., Wang X., Min H.-K., Dou W., Gong Z., Chan J., Dong W., Fan J.J. (2023). Mechanosensitive brain tumor cells construct blood-tumor barrier to mask chemosensitivity. Neuron.

[127] Yang H., Liu C., Zhou R.-M., Yao J., Li X.-M., Shen Y., Cheng H., Yuan J., Yan B., Jiang Q. (2016). Piezo2 protein: A novel regulator of tumor angiogenesis and hyperpermeability. Oncotarget.

[128] Huang Z., Sun Z., Zhang X., Niu K., Wang Y., Zheng J., Li H., Liu Y. (2019). Loss of stretch-activated channels, PIEZOs, accelerates non-small cell lung cancer progression and cell migration. Biosci. Rep..

[129] Lou W., Liu J., Ding B., Jin L., Xu L., Li X., Chen J., Fan W. (2019). Five miRNAs-mediated PIEZO2 downregulation, accompanied with activation of Hedgehog signaling pathway, predicts poor prognosis of breast cancer. Aging.

[130] Pardo-Pastor C., Rubio-Moscardo F., Vogel-González M., Serra S.A., Afthinos A., Mrkonjic S., Destaing O., Abenza J.F., Fernández-Fernández J.M., Trepat X. (2018). Piezo2 channel regulates RhoA and actin cytoskeleton to promote cell mechanobiological responses. Proc. Natl. Acad. Sci. USA.

[131] Katsuta E., Takabe K., Vujcic M., Gottlieb P.A., Dai T., Mercado-Perez A., Beyder A., Wang Q., Opyrchal M. (2022). Mechano-Sensing Channel PIEZO2 Enhances Invasive Phenotype in Triple-Negative Breast Cancer. Int. J. Mol. Sci..

[132] Liu X., Jia Y., Wang Z., Zhang Z., Fu W. (2022). A pan-cancer analysis reveals the genetic alterations and immunotherapy of Piezo2 in human cancer. Front. Genet..

[133] Pageon S.V., Govendir M.A., Kempe D., Biro M., Sawicka A., Babataheri A., Dogniaux S., Barakat A.I., Gonzalez-Rodriguez D., Hivroz C. (2018). Mechanoimmunology: Molecular-scale forces govern immune cell functions. Mol. Biol. Cell.

[134] Mihăilă R.-G. (2019). Liver stiffness in chronic hepatitis C virus infection. Rom. J. Intern. Med..

[135] Miyazawa A., Ito S., Asano S., Tanaka I., Sato M., Kondo M., Hasegawa Y. (2018). Regulation of PD-L1 expression by matrix stiffness in lung cancer cells. Biochem. Biophys. Res. Commun..

[136] Solis A.G., Bielecki P., Steach H.R., Sharma L., Harman C.C.D., Yun S., de Zoete M.R., Warnock J.N., To S.D.F., York A.G. (2019). Mechanosensation of cyclical force by PIEZO1 is essential for innate immunity. Nature.

[137] Sahoo S.S., Majhi R.K., Tiwari A., Acharya T., Kumar P.S., Saha S., Kumar A., Goswami C., Chattopadhyay S. (2019). Transient receptor potential ankyrin1 channel is endogenously expressed in T cells and is involved in immune functions. Biosci. Rep..

[138] Yin J., Michalick L., Tang C., Tabuchi A., Goldenberg N., Dan Q., Awwad K., Wang L., Erfinanda L., Nouailles G. (2016). Role of Transient Receptor Potential Vanilloid 4 in Neutrophil Activation and Acute Lung Injury. Am. J. Respir. Cell Mol. Biol..

[139] Talavera K., Startek J.B., Alvarez-Collazo J., Boonen B., Alpizar Y.A., Sanchez A., Naert R., Nilius B. (2020). Mammalian Transient Receptor Potential TRPA1 Channels: From Structure to Disease. Physiol. Rev..

[140] Jordt S.-E., Bautista D.M., Chuang H.-H., McKemy D.D., Zygmunt P.M., Högestätt E.D., Meng I.D., Julius D. (2004). Mustard oils and cannabinoids excite sensory nerve fibres through the TRP channel ANKTM1. Nature.

[141] Bautista D.M., Movahed P., Hinman A., Axelsson H.E., Sterner O., Högestätt E.D., Julius D., Jordt S.-E., Zygmunt P.M. (2005). Pungent products from garlic activate the sensory ion channel TRPA1. Proc. Natl. Acad. Sci. USA.

[142] Sawada Y., Hosokawa H., Hori A., Matsumura K., Kobayashi S. (2007). Cold sensitivity of recombinant TRPA1 channels. Brain Res..

[143] Moparthi L., Kichko T.I., Eberhardt M., Högestätt E.D., Kjellbom P., Johanson U., Reeh P.W., Leffler A., Filipovic M.R., Zygmunt P.M. (2016). Human TRPA1 is a heat sensor displaying intrinsic U-shaped thermosensitivity. Sci. Rep..

[144] Startek J.B., Talavera K., Voets T., Alpizar Y.A. (2018). Differential interactions of bacterial lipopolysaccharides with lipid membranes: Implications for TRPA1-mediated chemosensation. Sci. Rep..

[145] Shimizu S., Takahashi N., Mori Y. (2014). TRPs as Chemosensors (ROS, RNS, RCS, Gasotransmitters). Handb. Exp. Pharmacol..

[146] Horváth Á., Tékus V., Boros M., Pozsgai G., Botz B., Borbély É., Szolcsányi J., Pintér E., Helyes Z. (2016). Transient receptor potential ankyrin 1 (TRPA1) receptor is involved in chronic arthritis: In vivo study using TRPA1-deficient mice. Arthritis Res. Ther..

[147] Wechsler J.B., Hsu C.-L., Bryce P.J. (2014). IgE-mediated mast cell responses are inhibited by thymol-mediated, activation-induced cell death in skin inflammation. J. Allergy Clin. Immunol..

[148] Matsuda K., Arkwright P.D., Mori Y., Oikawa M.-A., Muko R., Tanaka A., Matsuda H. (2020). A Rapid Shift from Chronic Hyperoxia to Normoxia Induces Systemic Anaphylaxis via Transient Receptor Potential Ankyrin 1 Channels on Mast Cells. J. Immunol..

[149] Ma S., Wang D.H. (2021). Knockout of *Trpa1* Exacerbates Renal Ischemia–Reperfusion Injury with Classical Activation of Macrophages. Am. J. Hypertens..

[150] Wang Q., Chen K., Zhang F., Peng K., Wang Z., Yang D., Yang Y. (2020). TRPA1 regulates macrophages phenotype plasticity and atherosclerosis progression. Atherosclerosis.

[151] Huang W., August A. (2015). The signaling symphony: T cell receptor tunes cytokine-mediated T cell differentiation. J. Leukoc. Biol..

[152] Forni M.F., Domínguez-Amorocho O.A., de Assis L.V.M., Kinker G.S., Moraes M.N., de Lauro Castrucci A.M., Câmara N.O.S. (2021). An Immunometabolic Shift Modulates Cytotoxic Lymphocyte Activation During Melanoma Progression in TRPA1 Channel Null Mice. Front. Oncol..

[153] Szabó K., Kemény Á., Balázs N., Khanfar E., Sándor Z., Boldizsár F., Gyulai R., Najbauer J., Pintér E., Berki T. (2022). Presence of TRPA1 Modifies CD4+/CD8+ T Lymphocyte Ratio and Activation. Pharmaceuticals.

[154] Bertin S., Aoki-Nonaka Y., Lee J., de Jong P.R., Kim P., Han T., Yu T., To K., Takahashi N., Boland B.S. (2016). The TRPA1 ion channel is expressed in CD4+ T cells and restrains T-cell-mediated colitis through inhibition of TRPV1. Gut.

[155] Morty R.E., Kuebler W.M. (2014). TRPV4: An exciting new target to promote alveolocapillary barrier function. Am. J. Physiol.-Lung Cell. Mol. Physiol..

[156] Michalick L., Erfinanda L., Weichelt U., van der Giet M., Liedtke W., Kuebler W.M. (2017). Transient Receptor Potential Vanilloid 4 and Serum Glucocorticoid–regulated Kinase 1 Are Critical Mediators of Lung Injury in Overventilated Mice In Vivo. Anesthesiology.

[157] Scheraga R.G., Abraham S., Niese K.A., Southern B.D., Grove L.M., Hite R.D., McDonald C., Hamilton T.A., Olman M.A. (2016). TRPV4 Mechanosensitive Ion Channel Regulates Lipopolysaccharide-Stimulated Macrophage Phagocytosis. J. Immunol..

[158] Meli V.S., Atcha H., Veerasubramanian P.K., Nagalla R.R., Luu T.U., Chen E.Y., Guerrero-Juarez C.F., Yamaga K., Pandori W., Hsieh J.Y. (2020). YAP-mediated mechanotransduction tunes the macrophage inflammatory response. Sci. Adv..

[159] Scheraga R.G., Abraham S., Grove L.M., Southern B.D., Crish J.F., Perelas A., McDonald C., Asosingh K., Hasday J.D., Olman M.A. (2020). TRPV4 Protects the Lung from Bacterial Pneumonia via MAPK Molecular Pathway Switching. J. Immunol..

[160] Geng J., Shi Y., Zhang J., Yang B., Wang P., Yuan W., Zhao H., Li J., Qin F., Hong L. (2021). TLR4 signalling via Piezo1 engages and enhances the macrophage mediated host response during bacterial infection. Nat. Commun..

[161] Atcha H., Meli V.S., Davis C.T., Brumm K.T., Anis S., Chin J., Jiang K., Pathak M.M., Liu W.F. (2021). Crosstalk Between CD11b and Piezo1 Mediates Macrophage Responses to Mechanical Cues. Front. Immunol..

[162] Wang Y., Zhang Z., Yang Q., Cao Y., Dong Y., Bi Y., Liu G. (2022). Immunoregulatory Role of the Mechanosensitive Ion Channel Piezo1 in Inflammation and Cancer. Molecules.

[163] Liu C.S.C., Raychaudhuri D., Paul B., Chakrabarty Y., Ghosh A.R., Rahaman O., Talukdar A., Ganguly D. (2018). Cutting Edge: Piezo1 Mechanosensors Optimize Human T Cell Activation. J. Immunol..

[164] Jairaman A., Othy S., Dynes J.L., Yeromin A.V., Zavala A., Greenberg M.L., Nourse J.L., Holt J.R., Cahalan S.M., Marangoni F. (2021). Piezo1 channels restrain regulatory T cells but are dispensable for effector CD4^+^ T cell responses. Sci. Adv..

[165] Wang Y., Yang H., Jia A., Wang Y., Yang Q., Dong Y., Hou Y., Cao Y., Dong L., Bi Y. (2022). Dendritic cell Piezo1 directs the differentiation of TH1 and Treg cells in cancer. eLife.

[166] Aykut B., Chen R., Kim J.I., Wu D., Shadaloey S.A.A., Abengozar R., Preiss P., Saxena A., Pushalkar S., Leinwand J. (2020). Targeting Piezo1 unleashes innate immunity against cancer and infectious disease. Sci. Immunol..

[167] Mueller S., Sandrin L. (2010). Liver stiffness: A novel parameter for the diagnosis of liver disease. Hepatic Med. Evid. Res..

[168] Huse M. (2017). Mechanical forces in the immune system. Nat. Rev. Immunol..

